# The Arginine Decarboxylase Pathways of Host and Pathogen Interact to Impact Inflammatory Pathways in the Lung

**DOI:** 10.1371/journal.pone.0111441

**Published:** 2014-10-28

**Authors:** Nick B. Paulson, Adam J. Gilbertsen, Joseph J. Dalluge, Cole W. Welchlin, John Hughes, Wei Han, Timothy S. Blackwell, Theresa A. Laguna, Bryan J. Williams

**Affiliations:** 1 Pulmonary, Allergy, Critical Care and Sleep Division, University of Minnesota, Minneapolis, Minnesota, United States of America; 2 Department of Chemistry, University of Minnesota, Minneapolis, Minnesota, United States of America; 3 Division of Pediatric Pulmonology, University of Minnesota, Minneapolis, Minnesota, United States of America; 4 Division of Biostatistics, University of Minnesota, Minneapolis, Minnesota, United States of America; 5 Division of Allergy, Pulmonary, Critical Care and Sleep Medicine, Vanderbilt University Medical Center, Nashville, Tennessee, United States of America; University of North Dakota, United States of America

## Abstract

The arginine decarboxylase pathway, which converts arginine to agmatine, is present in both humans and most bacterial pathogens. In humans agmatine is a neurotransmitter with affinities towards α2-adrenoreceptors, serotonin receptors, and may inhibit nitric oxide synthase. In bacteria agmatine serves as a precursor to polyamine synthesis and was recently shown to enhance biofilm development in some strains of the respiratory pathogen *Pseudomonas aeruginosa*. We determined agmatine is at the center of a competing metabolism in the human lung during airways infections and is influenced by the metabolic phenotypes of the infecting pathogens. Ultra performance liquid chromatography with mass spectrometry detection was used to measure agmatine in human sputum samples from patients with cystic fibrosis, spent supernatant from clinical sputum isolates, and from bronchoalvelolar lavage fluid from mice infected with *P. aeruginosa* agmatine mutants. Agmatine in human sputum peaks during illness, decreased with treatment and is positively correlated with inflammatory cytokines. Analysis of the agmatine metabolic phenotype in clinical sputum isolates revealed most deplete agmatine when grown in its presence; however a minority appeared to generate large amounts of agmatine presumably driving sputum agmatine to high levels. Agmatine exposure to inflammatory cells and in mice demonstrated its role as a direct immune activator with effects on TNF-α production, likely through NF-κB activation. *P. aeruginosa* mutants for agmatine detection and metabolism were constructed and show the real-time evolution of host-derived agmatine in the airways during acute lung infection. These experiments also demonstrated pathogen agmatine production can upregulate the inflammatory response. As some clinical isolates have adapted to hypersecrete agmatine, these combined data would suggest agmatine is a novel target for immune modulation in the host-pathogen dynamic.

## Introduction

The human lung is normally a sterile environment given numerous highly evolved mechanisms to capture, destroy and remove inhaled pathogens [1]. Defects in these mechanisms, be it inherited or acquired, may lead to persistent infections of the airways resulting in chronic bronchitis and bronchiectasis. The persistently infected lung is usually characterized by damaged airways harboring purulent sputum. This sputum contains a rich mixture of metabolites, namely amino acids, peptides, and nucleic acids spilled from neutrophils that have been recruited to fight the airways infection [2]. This rich environment drives bacterial densities to very high levels; up to 10^9^ colony forming units (cfu) per mL. Despite the abundance of nutrients, a large proportion of the bacteria in these airways are not rapidly dividing planktonic organisms but embedded in a biofilm [3]. Bacterial biofilms have been observed in diseased lungs of patients with chronic obstructive pulmonary disease, cystic fibrosis (CF), and other forms of bronchiectasis [4], [5]. In the laboratory, bacterial biofilms are characterized by adherence to a surface, slower growth rates, and nutrient limitation [6]. However in the bronchiectatic airway, the bacteria are not adhered to a cell surface and not apparently limited in nutrients, thus other environmental cues found in the matrix of human sputum must trigger these bacteria to grow as a biofilm [7], [8].

Amino acids are found in sputum in millimolar quantities, and are the key energy source for the bacteria found there [9]. *Pseudomonas aeruginosa*, a well-studied cause of persistent lung infections, quickly adapts its metabolic profile upon entering the lung to one of amino acid utilization. Arginine plays a pivotal role in a number of *P. aeruginosa*'*s* metabolic pathways, particularly in oxygen limiting environments, including the lung [10], [11]. A key pathway for arginine utilization in *P. aeruginosa* is the arginine decarboxylase pathway which converts arginine into the pre-polyamine agmatine [12]. Pseudomonal derived agmatine is quickly converted into the polyamines by the agmatine deiminase pathway (*aguBA*) [13]. The arginine decarboxylase pathway also exists in mammals and agmatine has been associated with higher order functions such as a neurotransmitter and regulation of nitric oxide through competition with arginine [14], [15]. Agmatine has been measured in human serum at a value of ∼400 nM [16], and its production may increase during sepsis [17]. It has been measured in animal tissues and found to be present in whole lung homogenates, but this does not exclude the contribution of serum agmatine [18]. Finally, agmatine is a known α2-adrenoreceptor and imidazoline receptor agonist with suggested roles including vasodilation and the prevention of opioid induced tolerance [19], [20].

We recently discovered an alternate operon in *P. aeruginosa* (*agu2ABCA*') that appears to detect and respond to environmental agmatine [21]. This operon was preferentially expressed when *P. aeruginosa* was growing as a biofilm and enhanced the biomass of a biofilm in the presence of agmatine. Without *agu2ABCA*', agmatine appeared to inhibit biofilm formation. This suggests agmatine, normally a preferred metabolite for *P. aeruginosa*, may be an environmental trigger for biofilm formation in the lung. In this work we determine that agmatine is not only present in human sputum, but is both associated with and capable of causing inflammation. Given the presence of arginine decarboxylase pathways in both prokaryotes and eukaryotes, we investigated the source of agmatine production in sputum and determined both host and pathogen influences are likely active concomitantly. Using a luminescent pseudomonal reporter of agmatine, we show that host agmatine production is detected by the infecting organisms. Furthermore we demonstrate that bacterial excretion of agmatine, as seen in some naturally occurring clinical isolates, may enhance the host inflammatory response. Together, these data uncover a new mechanism of host-pathogen interaction through shared recognition of agmatine and implicates competing agmatine metabolic pathways in both inflammatory responses and bacterial biofilm formation simultaneously.

## Results

### Agmatine is found in the lung and associated with illness and inflammation

**Figure 1:**
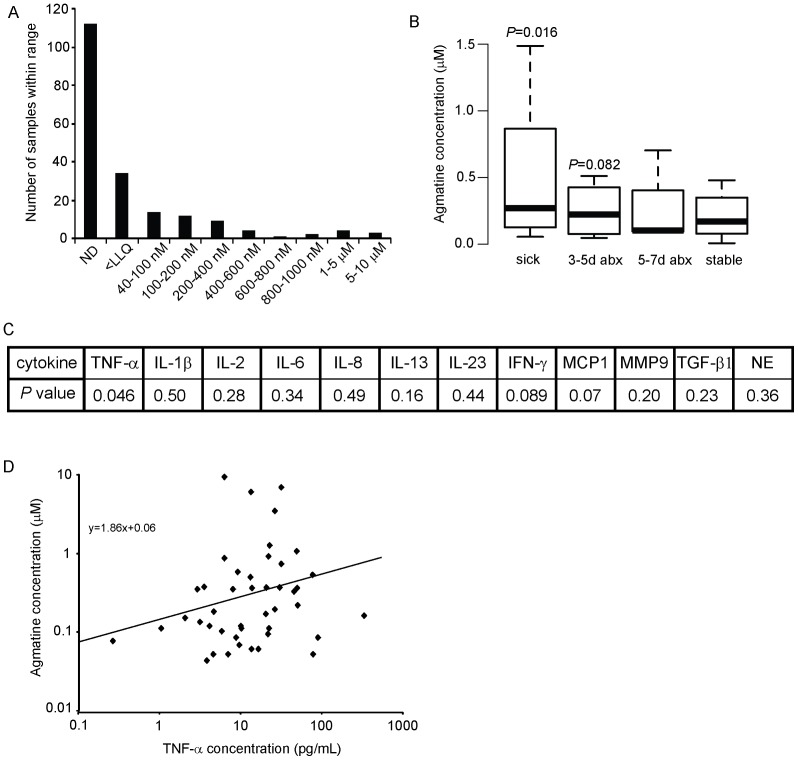
Sputum agmatine measurements. (A) Distribution of agmatine concentration in 197 human sputum samples as measured by UPLC-MS/MS. (B) Sputum agmatine values during illness. Medians, 1st and 3rd quartiles and range within 1.5 times the quartiles shown by thick bars, boxes and whiskers respectively. One way ANOVA performed across timepoints with significance shown. *P* values are compared to stable and were adjusted for multiple comparisons. (C) Sputum cytokines were compared to sputum agmatine (in samples with quantifiable agmatine). NE-neutrophil elastase. (D) Sputum agmatine and TNF-α value correlation. See methods for statistical approach to non-normal data.

Through the recently discovered *agu2ABCA*' operon, *P. aeruginosa* has a mechanism to detect extracellular agmatine and react by augmenting its biofilm [21]. This suggests *P. aeruginosa* may encounter agmatine in lung infections, and that this may trigger planktonic pseudomonads to form a biofilm. Sputum from patients with CF was tested for various cytokines by ELISA and also agmatine using ultraperformance liquid chromatography tandem mass spectrometry (UPLC-MS/MS). These sputum samples were generated from patients who were considered to be at baseline regarding their lung symptoms, having a pulmonary exacerbation with an increase in symptoms, or during treatment with antibiotics for their lung infections. The measured range in sputum samples demonstrates most were below the detection limit of our assay of 40 nM (Figure 1A), however a large concentration range is present, possibly demonstrating a dichotomous distribution with very high levels (some >1 µM) and undetectable levels. Evaluating the disease state present when samples with measurable agmatine were expectorated reveals agmatine is higher during sickness and initial antibiotic use and decreases with treatment (Figure 1B). Correlating agmatine to sputum cytokines demonstrates a statistically significant correlation between agmatine concentration and TNF-α and trends towards significance with MCP-1, and IFN-γ (Figures 1C and D). These data suggest agmatine may be associated with illness and inflammation, and coupled with its known biologic activity as a receptor agonist, prompted us to explore its potential role as an inflammatory mediator.

### The origin of airway agmatine during infection

The CF sputum data demonstrates agmatine is found in the airways, but it is not clear if this agmatine is of a bacterial or eukaryotic source. Agmatine has been measured with various analytical chemistry methods from multiple organs in mice and humans, and its level varies greatly from system to system [18]. Furthermore, arginine decarboxylase has been shown to be upregulated from the macrophage-like cell line RAW 264.7 in response to LPS and cytokines resulting in more intracellular agmatine [22]. We attempted to measure agmatine from the intracellular and extracellular compartments of primary mouse macrophages, human peripheral blood neutrophils, and human bronchial cell lines (BEAS-2B), but failed to measure agmatine in any of these situations, including LPS stimulation (data not shown). Many bacteria create agmatine in the path to produce polyamines from arginine, but it is not clear if agmatine is actively secreted by many bacteria [23]. We have measured both extracellular and intracellular agmatine in the model *P. aeruginosa* PA14 and have found that agmatine is synthesized by the arginine decarboxylase pathway, but is essentially undetectable if the *aguBA* operon is left intact [21].

**Figure 2:**
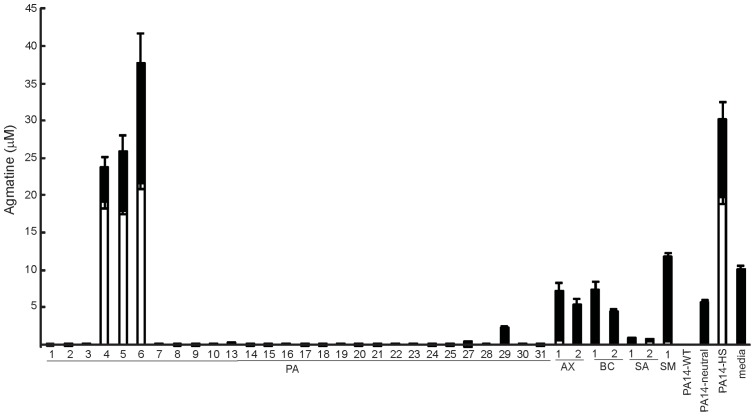
Agmatine metabolic phenotype of clinical bacterial isolates from sputum. Supernatant agmatine was measured by UPLC-MS/MS after 24 h growth in RPMI without (white bars) or with (black bars) 10 µM agmatine. PA- *P. aeruginosa*, AX- *Achromobacter xyloxidans*, BC- *Burkholderia cepacia*, SA-*Staphylococcus aureus*, SM- *Serratia marcesens*, PA14-WT *P. aeruginosa* laboratory strain used in these studies, PA14-neutral mutant with genotype Δ*speA*, *aguA:gm*, Δ*agu2ABCA*' neither creates nor degrades agmatine, PA14-hypersecretor mutant with genotype *aguA::gm*, *Δagu2ABCA*', creates but cannot degrade agmatine. Bars represent average measured values of triplicate analyses by UPLC-MS/MS. Error bars represent SEM.

To determine if bacterial agmatine metabolism could contribute to the agmatine pools found in human sputum, we analyzed a panel of clinically-derived bacterial isolates representing most of the predominant species found in CF sputum. These isolates were tested for their ability to secrete or destroy agmatine (Figure 2). Most of the *P. aeruginosa* isolates and the *S. aureus* isolates studied do not secrete agmatine but rather depleted supplemental agmatine after overnight growth in liquid culture. However, three *P. aeruginosa* isolates were found that hypersecrete agmatine to levels that closely resemble that seen in the PA14 mutant of *aguBA* and *agu2ABCA*' that secretes agmatine as it cannot utilize it. The presence of agmatine hypersecretors suggested the sputum samples with the highest agmatine concentration may have been the result of bacterial agmatine secretion, and those with undetectable agmatine may have been subject to bacterial depletion. While bacteria were not initially cultured from the sputum sample set used for agmatine analysis we attempted to recover bacterial isolates from the frozen sputum samples with the highest agmatine values and were successful in recovering one pseudomonad that shared this same hypersecretion phenotype (data not shown). *B. cepacia* and *A. xyloxidans* appear to be “agmatine neutral” having no effect on agmatine concentrations in liquid culture.

**Figure 3:**
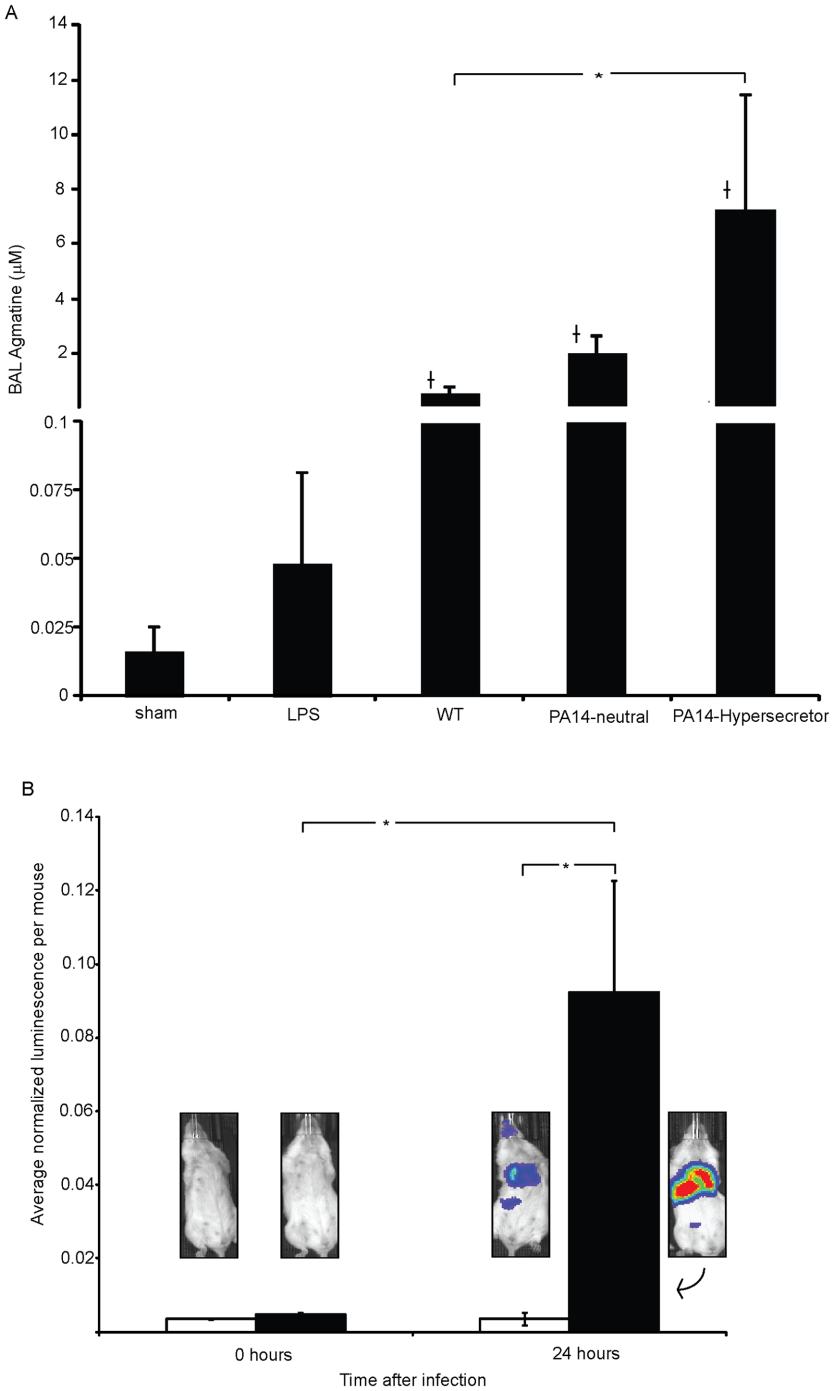
Host agmatine response and detection by *P.aeruginosa in vivo*. (A) Agmatine was measured by UPLC-MS/MS in bronchoalveolar lavage (BAL) fluid collected 24 h after no treatment, treatment with LPS, or infection with the *P. aeruginosa* mutants shown on the x axis (mutants described in Figure 2). 3 mice were used in the LPS and sham groups and 10 mice used in the *P. aeruginosa* groups. (B) Bacterial detection of agmatine was determined using bioluminescent reporter strains as described in the text. The reporter strain (filled bar) contains the *aguR*-*B* response element fused to the *luxCDABE* operon. The control strain (unfilled bar) is identical but does not include the transcriptional element. Infected mice were imaged using *in vivo* animal imaging immediately after inoculation, or just before sacrifice at 24 hours. Each bar represents the average relative luminescence normalized to the actual inoculum at time 0 or the cfu of the total lung volume at 24 hours after imaging. Photo insets show representative mouse images from groups corresponding to data bars. All error bars represent SEM. Paired t-test used to compare mice in same groups, and an independent t-test was used for mice infected with different strains. *P*<0.05 compared to LPS or sham groups. *P<0.05.

To determine if the lung could serve as a source of agmatine during infection we exposed mouse lungs to LPS and various *P. aeruginosa* mutants of agmatine metabolism and measured the agmatine found in bronchoalveolar lavage (BAL) fluid by UPLC-MS/MS (Figure 3A). The BAL fluid demonstrates the presence of agmatine at baseline, increasing levels with LPS treatment, but dramatically higher levels with bacterial infection. Furthermore the bacterial pathways of agmatine metabolism are able to impact the agmatine levels within the lung during infection. Wild-type *P. aeruginosa*, which is able to actively consume agmatine produces the lowest signal. The agmatine hypersecretor mutant (*aguA:gm*, Δ*agu2ABCA*'), which secrete agmatine similarly to the clinical isolates in Figure 2, produces the highest signal. The agmatine neutral mutant which neither produces nor destroys agmatine (Δ*speA, aguA:gm, Δagu2ABCA*') reveals the “un-altered” level of agmatine produced by the lung in response to the bacterial infection.

To demonstrate that this mouse-derived agmatine could be detected by infecting bacteria the agmatine neutral mutant was also engineered to contain an agmatine responsive bioluminescent reporter by fusing the promoter and beginning coding sequence of the *aguBA* operon into a single copy, genomically-integrated *lux* operon [24]. This mutant produces light in a dose dependent fashion when exposed to extracellular agmatine whereas its “empty vector” control strain does not (Figure S1). The light output of the infecting mutant was measured and normalized to either the infecting inoculum at time zero or the recovered bacterial colony count from the BAL (Figure 3B). The reporter demonstrates a significantly higher light output over the chest during pneumonia than does the vector control reporter that does not respond to agmatine. This demonstrates bacterial detection of host agmatine during lung infections. Combined, these experiments suggest that sputum agmatine concentrations could be derived from either the host lung or, in some instances, the bacteria themselves, and are subject to bacterial manipulation.

### Agmatine's effects on the immune response

**Figure 4:**
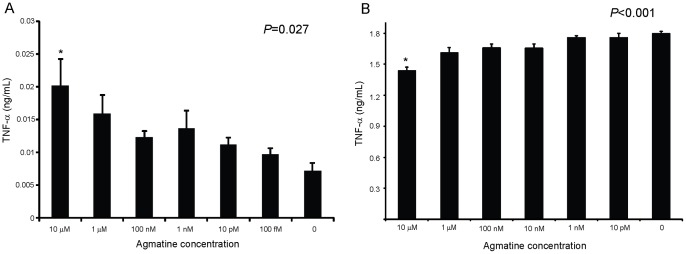
Agmatine's effects on TNF-α production in macrophages. (A) Mouse peritoneal macrophages exposed to agmatine titration for 24 h and cell supernatant TNF-α measured by ELISA. (B) Same as (A) except cells also treated with 10 ng/mL LPS. 4–5 wells per concentration were assayed. One way ANOVA performed with significance shown above each figure. All error bars represent SEM. Tukey's post-hoc analysis used to determine significance for specified concentration compared to 0. *P<0.05.

As agmatine is positively correlated with inflammation in human sputum, we sought to determine if agmatine could induce an inflammatory response. Macrophages and neutrophils are the key white blood cell determinants of the immune response in the lung and to *P. aeruginosa* [1]. Agmatine's role in the induction or the manipulation of their cytokine responses has not been studied. Agmatine demonstrated a dose effect on TNF-α production in primary murine peritoneal macrophages (Figure 4A). A similar trend was observed with MIP-2a (murine version of human IL-8) suggesting the TNF-α response is mediated by NF-κB (data not shown). As sputum from patients with cystic fibrosis represents a chronic infection, it is unlikely agmatine will act on naïve cells free of other co-stimulants. When the agmatine titration in macrophages was repeated with LPS co-stimulation, a reversal in effect with an inhibition of TNF-α in LPS stimulated cells was observed (Figure 4B). This suggests agmatine is capable of both immune activation and inhibition dependent on dose, and the presence of co-stimulatory molecules. Furthermore, the effective dose range is within the true biologic range measured in sputum (Figure 1A) suggesting these immunomodulatory effects may occur within the more complex environment of the lung.

**Figure 5:**
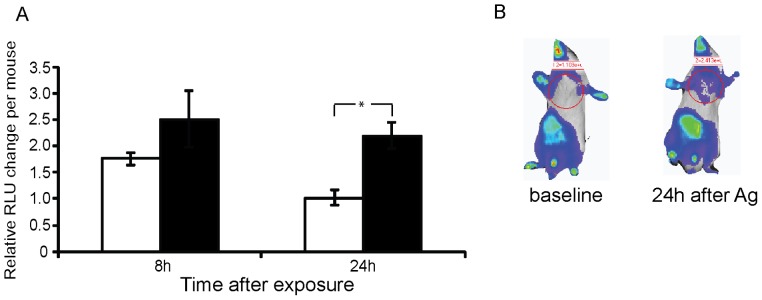
Agmatine induces NF-κB in animal models of inflammation. HLL NF-κB reporter mice were challenged intratracheally with PBS (unfilled) or agmatine (filled) and luminescence was measured at the specified timepoints after injection as described in the methods. Each mouse served as its own control and the relative change per mouse compared to background luminescence is plotted on the y-axis. In (A) there are 3 mice in the PBS group and 5 mice in the Ag group and luminescence was measured over the chest only as shown in (B). This experiment was replicated on 2 other occasions with similar results. Panel (B) shows representative images of individual mice in these studies. Independent t-tests were used between groups of mice with relevant comparisons shown. All error bars represent SEM. Independent t-tests used between groups. *P<0.05.

To determine which of these effects occur within the multicellular environment of a mammal we utilized a mouse model of real time inflammation. The HLL mouse contains the gene for luciferase fused to an NF-κB response element [25]. Thus cellular expression of NF-κB in a live animal can be monitored by luminescence output after administration of systemic luciferin using an animal imaging station. Agmatine was intratracheally injected into the lungs of these NF-κB reporter mice and luminescence over the lung field was measured revealing a significant increase in lung NF-κB expression at 24 hours compared to PBS injection alone (Figures 5A and B). While there is an upregulation of NF-κB with intratracheal administration of agmatine, there is not a significant injury to the lung when viewed on histopathology, nor is there a significant increase in cells recruited to the alveoli when measured in bronchoalveolar lavage fluid at 24 hours (data not shown). The *in vitro* macrophage data (Figure 4A) suggests the administered agmatine may have stimulated the resident cells of the lung (macrophages or epithelial cells) without inducing a measurable recruitment of neutrophils. In an attempt to replicate the co-stimulatory conditions of the cell culture experiments we intratracheally injected both LPS and agmatine into the lungs of mice. However the inflammatory response over the lungs alone was difficult to measure given the robust systemic response to LPS in the liver and abdomen (data not shown). Using a similar NF-κB reporter mouse (NGL) we administered both LPS and agmatine via the intraperitoneal route and measured the total body NF-κB response in this model (Figure S2A and B). At 4 hours agmatine augments the LPS induced NF-κB response, but this response is more rapidly diminished by 8 hours. As with the cellular response, the systemic response to agmatine and LPS in an animal model is likely complex, however it is clear that agmatine administration does augment the inflammatory response *in vitro* and *in vivo* when exogenously administered.

### Bacterial metabolism of host agmatine alters the inflammatory response

**Figure 6:**
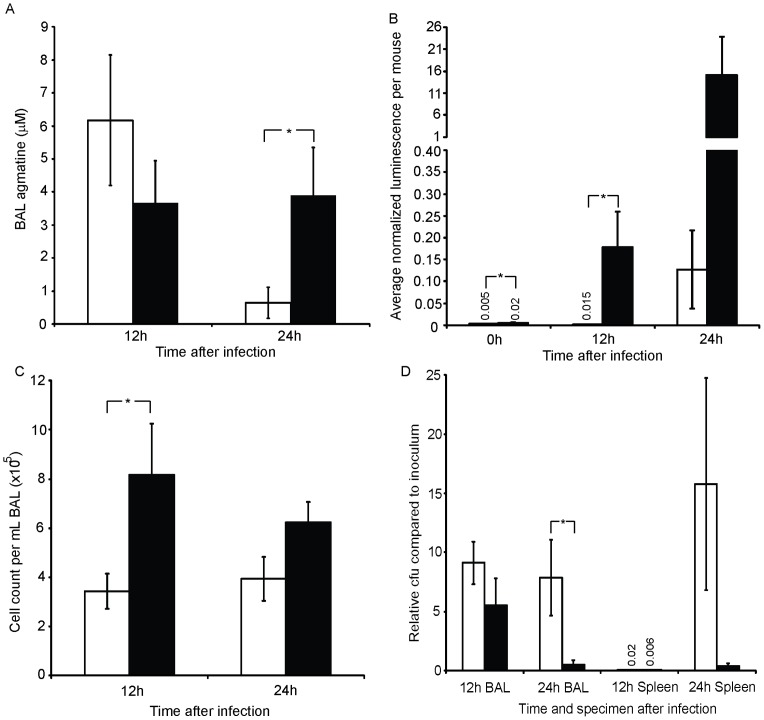
Bacterial agmatine secretion alters inflammation and bacterial survival. *P. aeruginosa* strain PA14 (unfilled bars), and an agmatine metabolism mutant (filled bars, *aguA::gm, Δagu2ABCA*') were injected at 1×10^6^ cfu per mouse. Both harbored the *aguR-B:lux* reporter system. 10 mice injected per group per timepoint. The agmatine metabolism mutant strain hypersecretes agmatine similarly to the clinical isolates shown in Figure 2. In (A), the average BAL agmatine, as measured by UPLC-MS/MS, is plotted for both groups at each timepoint. In (B) the bacterial luminescence during infections was measured as an indicator of *in vivo* agmatine secretion/detection by the bacterial *aguR:lux* system. The luminescence is normalized to the cfu count obtained shortly after imaging. At the times indicated mice were sacrificed for bronchoalveolar lavage and spleen removal for measurement of inflammatory cell count (C), and bacterial cfu counting (D). All error bars represent SEM. Independent t-tests used between groups. *P<0.05.

As agmatine is found in the sputum of patients with chronic infection, and appears capable of inducing an inflammatory response, we hypothesized that bacterial metabolism of agmatine during an infection could alter the host inflammatory response. As demonstrated above (Figure 2A), most of the pseudomonads found in cystic fibrosis lungs are capable of metabolizing agmatine in a closed system to exhaustion, with the exception of some mutants that hypersecrete agmatine. Furthermore bacterial mutants replicating these phenotypes were able change the agmatine found in the lung during infection (Figures 3A and B). To determine if bacterial manipulation of agmatine in the airways could alter the inflammatory response, we infected mice with WT strain PA14 and the hyper-secretor mutant strain (*aguBA* and Δ*agu2ABCA*', Figures 6A–D). Both of these strains harbored the *aguB:luxBCADE* reporter construct as well. BAL agmatine was significantly higher in the mutant group by 24 h (Figure 6A) suggesting the mutant contributes agmatine to the lung milieu and the bacterial reporter construct demonstrates more agmatine detection per bacteria by the mutant strain (Figure 6B). The impact of bacterial agmatine secretion was evident in changes in the inflammatory phenotype including increased total cell count (Figure 6C). While there is not growth defect *in* vitro between these two bacterial strains [21], there is a survival difference (Figure 6D), possibly due to the increased cellular response in the mice infected with the hyper-secretor mutant. BAL TNF-α was also measured in these groups and was found to be lower in mice infected with the hypersecretors, which may be the result of decreased bacterial burdens (data not shown). This suggests the immune upregulation induced by agmatine seen in cell culture, and animal models, is also at play during bacterial infection, but clearly under the influence of bacterial metabolism. These data establish a role for the bacterial arginine decarboxylase pathway as host immune modulator.

## Discussion

With advances in analytical techniques, the ability to track multiple small molecules in diverse matrices has led to a heightened appreciation of the complicated chemical mediators of both immune cell and bacterial signaling. Frequently these signaling molecules, such as cytokines and quorum sensing molecules, are unique to a species, having the presumed intent of communicating a very specific message to neighboring cells. Occasionally a pathogen may adapt a way to intercept or destroy cell signaling molecules with potential benefit to bacterial survival [26], [27]. This work on the arginine decarboxylase pathways of mammals and bacteria was spawned by the observation that the benign molecule agmatine induces select *P. aeruginosa* strains to form a biofilm. Agmatine has no deleterious effect on *P. aeruginosa* up to millimolar quantities, and is readily metabolized to putrescine which can be a source of ATP production after conversion to alanine or succinate [28], [29]. While many of the cues that coerce a pathogen to form a biofilm are not known, most are thought to be cues of environmental stress. This suggests that agmatine may be a cue of stress to *P. aeruginosa* in one of its natural environments.

Agmatine has not been described in the human lung until now. Its role in human biology is poorly understood having only recently been shown to exist in mammals [15], [30]. It has known receptor affinities for α2-adrenoreceptors, serotonin, and imidazoline receptors, and has been shown to be a direct inhibitor to NOS-2 presumably given its similarity to the NOS substrate arginine [30]. It is not known how important agmatine is in most organ systems, or if its receptor actions are evolutionarily intended or merely a consequence of similarity to the known ligands of each of those receptors. Our data suggest agmatine does have a direct impact on inflammation with TNF-α induction in macrophages and NF-κB induction in mouse lungs. A potential mode of action is through the α2-adrenoreceptors which have been shown to exist on macrophages and neutrophils and are important in lung inflammation [31]. Catecholamines have been shown to be ligands of adrenoreceptors on neutrophils, which are capable of norepinephrine secretion for a local autocrine signaling upon LPS stimulation. We could not detect agmatine secretion at baseline or after stimulation in macrophages so it would not appear that agmatine and catecholamines have overlapping purposes, although its functional range could be below the detection limit of our UPLC-MS/MS system. Furthermore, agmatine appears to have an inhibitory effect in the presence of LPS suggesting either a different receptor action is predominant or the receptor signaling has reversed its mode of action as previously described in α2-adrenoreceptors [32]. The cellular origin of mammalian agmatine in the lung remains unknown but is clearly induced by LPS or bacterial infection. It is possible that agmatine is from the vascular space and spilled during infection with *P. aeruginosa* as blood contains ∼400 nm agmatine [16]. If this is true, it might indicate that agmatine signaling in the lung serves as a paracrine message of nearby hemorrhage, or a danger signal. The animal studies also suggest the immunomodulatory effects of agmatine are not limited to the lung as intraperitoneal injection of agmatine also skewed the abdominal NF-κB response. Studies to determine the evolution of mammalian agmatine, its receptors and modes of actions that translate into TNF-α production are currently underway in our laboratory.

Cell death and lysis is a well-established source of much of the matrix found in CF sputum and a number of studies have shown pathogens respond to numerous molecular cues found there [7]. As *P. aeruginosa* harbors one of the largest metabolically capable genomes known, it is not surprising that it contains the genetic machinery to utilize exogenous agmatine [33]. We previously demonstrated that 100% of a panel of *P. aeruginosa* clinical isolates from various infectious sites contained the *aguBA* operon for conversion of agmatine to putrescine [21]. This work shows most sputum isolates exhaust exogenously supplied agmatine to levels below our detection limit by UPLC-MS/MS. However, a small percentage of our isolates appear to be agmatine metabolic mutants as they secrete agmatine to high levels. We have isolated one of these agmatine hypersecretors from a sputum sample with a very high agmatine concentration, suggesting *P. aeruginosa* can also contribute to the agmatine in the human airways when these mutants are present. Using lab-created mutants that mimic these hypersecretors we determined that bacterially produced agmatine could increase the airway agmatine balance and the inflammatory response. As most *P. aeruginosa* in our clinical panel consume agmatine, this should have the net effect of reducing inflammation, possibly allowing the bacteria to thrive. And while agmatine hypersecretion appears to reduce bacterial cfu in the acute pneumonia model, the presence of these mutants in patients with chronic infections suggests there may be a biologic benefit of agmatine secretion and inducing an inflammatory response. As *P. aeruginosa* grows in a biofilm in these chronic infections, they are typically resistant to the actions of neutrophils, but may derive most of their metabolites from dead neutrophils. As *P. aeruginosa* clones can persist for decades in CF airways, tracking the behavior of an agmatine-hypersecretor in a patient's lung over time and correlating this with clinical outcomes may suggest a reason to retain this mutation in the lung.

These results establish a new role for the pre-polyamine agmatine in the lung, and potentially in other niches throughout the human body. As agmatine is also an important bacterial metabolite that can be manipulated during an infection, a new precedent in the host-pathogen dynamic has been established.

## Materials and Methods

### Ethics statement

The University of Minnesota Institutional Review Board approved all human studies mentioned in this manuscript. All patients that provided sputum samples for this work were adults and provided written informed consent. The vertebrate animal work in this manuscript followed the “Guide for the Care and Use of Laboratory Animals” published by the Association for Assessment and Accreditation of Laboratory Animal Care (AAALAC). The University of Minnesota Institutional Animal Care and Use Committee (IACUC) has approved our experimental protocols involving vertebrate animals (protocol ID number 1002A77437) and is accredited by the AAALAC and follows the NIH Welfare Guidelines (Assurance number A3456–01, expires April 30, 2016).

### Sputum collection and analysis

Agmatine was measured in sputum samples collected for another study at our institution. This was a single-center, prospective, two-year longitudinal cohort study of patients with CF during times of pulmonary exacerbation (hospitalization) and times of clinical stability (outpatient clinic visits). For the purposes of this study, a pulmonary exacerbation was defined as the need for hospitalization for intravenous (IV) antibiotics and aggressive airway clearance for an increase in pulmonary symptoms (i.e. cough or sputum production), a >10% decrease in forced expiratory volume after 1 second compared with baseline and/or attending physician clinical judgment. The study protocol was approved by the University of Minnesota Institutional Review Board and informed consent and/or assent were obtained from each of the subjects. Each subject provided an expectorated sputum sample on enrollment with an illness, day 3–5 of therapy, day 5–7 of therapy, and after completing antibiotics during any visits when not ill. Sputum was processed as previously described and frozen immediately after collection at −80°C prior to analysis [34]. The proteases (neutrophil elastase and matrix metalloproteinase [MMP-9]) were measured in thawed untreated specimens. Free neutrophil elastase activity was quantified by a spectrophotometric assay based on the hydrolysis of the specific substrate MeO-suc-Ala-Ala-Pro-Ala-*p*-nitroanilide (Sigma Chemical Co, St. Louis, MO) whereas MMP-9, IL-23, and TGF-β were measured using commercially available ELISA kits (R&D Systems, Minneapolis, MN). IFN-γ, IL-1β, IL-2, IL-6, IL-8, MCP-1, and TNF-α were analyzed as an eight-plex (EMD Millipore Corporation, Billerica, MA).

### Analysis of agmatine in biologic substances

A Waters Acquity UPLC/triple quadrupole mass spectrometer (Waters, Milford, MA) was used for determination of agmatine. A Waters HSS T3 2.1 mm ×100 mm column (1.7 μm particles) at 35°C was used during the following 10 min gradient separation with A: water containing 0.1% formic acid and B: ACN containing 0.1% formic acid, at a flow rate of 0.4 mL/min: 3% B, 0 min to 2.0 min; 3% B to 48% B, 2.0 min to 3.0 min; 48% B 3.0 min to 5.0 min; 48% B to 97% B, 5.0 min to 5.5 min; 97% B, 5.5 min to 7.5 min; 97% B to 3% B, 7.5 min to 7.8 min; and 3% B, 7.8 min to 10.0 min. By directly infusing agmatine and ^13^C_5_,^15^N_4_-agmatine, cone voltages and collision energies for each selected reaction monitoring (SRM) transition were optimized. The transitions that produced the highest sensitivity for the determination of each analyte were selected for quantification: Agmatine: 294.2 to 235.0; ^13^C_5_,^15^N_4_-agmatine: 303.2 to 240.1. Dwell time for each transition was 0.05 s. For electrospray ionization tandem mass spectrometry (ESI-MS/MS) in positive ionization mode, parameters were as follows: capillary, 3.5 kV; cone, 40 V; extractor, 3 V; rf lens, 0.3 V; source temperature, 100°C; desolvation temperature, 350°C; desolvation flow, 1000 L/h; cone gas flow, 20 L/h; low-mass resolution (Q1), 15 V; high-mass resolution (Q1), 15 V; ion energy (Q1), 0.3 V; entrance −5 V; exit, 1 V; collision energy 20 V; low-mass resolution (Q2), 15 V; high-mass resolution (Q2), 15 V; ion energy (Q2) 3.5 V.

For standardization, 8 levels of calibration mixtures ranging from 0 ng/mL to 10,000 ng/mL were prepared for agmatine and ^13^C_5_,^15^N_4_-agmatine to achieve 8 different response ratios for agmatine in the mixtures. These solutions were then analyzed by UPLC-MS/MS, and the data were subjected to a linear least squares analysis. The peak area ratios of analyte:internal standard measured in samples (prepared as described below) spiked with a fixed relative amount of internal standard equal to that present in the standard solutions were then used in conjunction with the calibration curves to determine the concentration of agmatine in the samples. Limits of detection (LOD) and quantitation (LOQ) were calculated by determining the signal-to-noise values for samples spiked with 50 ng/mL agmatine and extrapolating to the concentration at which the signal-to-noise value was 10 for LOQ or 3 for LOD.

Sample preparation was as follows: 100 µL of sample spiked with internal standard was mixed with 200 µL of ice cold isopropanol and chilled to −20°C for 5–8 h. The sample was centrifuged at 21,000×g and the supernatant separated from the proteinacious pellet to a Amicon Ultra 3 kDa MW cutoff filtration column (Millipore). This column was centrifuged at 14,000×g for 4–6 hours to recover at least 100 mL of filtrate. To 100 µL of filtrate, 15 µL of borate buffer (pH 9.5) was added, followed by 15 µL of 10 mM NBD-F (Sigma) in acetonitrile. The sample was mixed and placed at 60°C for 10 min. After incubation the sample was placed on ice, then treated with 20 mL of 0.3% formic acid within 2 min to stabilize the NBD-derivatized analytes. The sample was centrifuged for 5 min at 21,000×g, and the supernatant centrifuged through an Ultrafree-MC GV filter column (Millipore) for final particulate removal prior to analysis by UPLC-MS/MS.

### Clinical isolate collection and bacterial growth

The clinical microbiology lab at the University of Minnesota Medical Center hospital isolated and identified pathogenic isolates from the sputum of patients with CF. These samples were collected separately from those sputum samples used in agmatine analysis. *P. aeruginosa* strain PA14 [33], its agmatine mutants, and the *P. aeruginosa* clinical isolates were either grown in Luria Bertani media or RPMI media as indicated in the text. All growth occurs at 37°C with orbital shaking for liquid cultures at 225 rpm. For UPLC-MS/MS analysis of spent supernatant RPMI was used as it is a defined medium without added agmatine and did not suffer the same analyte suppression as LB.

### Bacterial mutagenesis

The *speA* gene was amplified from *P. aeruginosa* PA14 genomic DNA using forward primer 5′-TTGTTGACCTGGCCCGTCGA-3′ and reverse primer 5′-GGGAAGCGGAAATGAAGGGG-3′ and inserted into pEX18 Amp utilizing the native *Eco*RI and *Hind*III sites within the PCR fragment [35]. To create the *speA* knockout the plasmid was digested with *Sph*I and *Eco*RV, followed by conversion to blunt ends. This removed 744 bp near the center of *speA* leaving flanking regions of 1035 bp and 842 bp on either side. The final construct was transformed into the mating *E. coli* strain SM10 and subsequently mated with PA14 or the agmatine pathway mutant strains. The genomic DNA of the resulting mutants was screened via PCR using the primers for *speA* amplification. The loss of agmatine production by *speA* mutants was validated by loss of agmatine production as assessed by UPLC-MS/MS. All PCR reactions were performed with the GC-Rich PCR system (Roche, Indianapolis, IN). Mutations in PA14 for *aguA*, and the *agu2ABCA*' operon were described previously [21].

The luminescent reporter construct was created by inserting the *aguBA* transcriptional element into the mini-ctx-lux vector as previously described [24]. Before use, the t7 promoter upstream of the multiple cloning site in mini-ctx-lux was removed by site directed mutagenesis (Mutagenex, Piscataway, NJ) to reduce background luminescence. The *aguR-B* fragment was amplified from PA14 genomic DNA using forward primer 5′-GCAAGCTTTGGCGTCCAATAGCCGCTCAC-3′ and reverse primer 5′-GCGAATTCAGTTCCTGGATCAGGATGATCTGC-3′. The forward primer includes a *Hind*III site and the reverse primer includes a *Eco*R1 site which were used to clone the PCR fragment into the mini-ctx-lux vector. All analyses of the agmatine reporter construct were compared to identical mutants with the mini-ctx-lux vector alone to establish background luminescence. Plate based luminescence was measured in a SpectraMax M3 spectrophotometer (Molecular Devices, Sunnyvale, CA).

### Expression of *speA* and synthesis of isotopic agmatine

The gene for *speA* was amplified from PA14 genomic DNA using forward primer 5′–CACCATGGCCGCTCGACGGACT-3′ and reverse primer 5′-GGACAGGTACGCCGAGCGG-3′ and then cloned into the pBAD Directional TOPO vector as described by the manufacturer (Life technologies, Green Island, NY). The SpeA gene product was expressed and purified per manufacturer instructions. The purified protein was reacted with a 10 mM concentration of L-Arginine-^13^C_6_, ^15^N_4_ hydrochloride (Sigma), 100 mM HEPES pH 8.4, 5 mM MgSO_4_, 1 mM DTT, 0.04 mM pyridoxal phosphate at 37°C for 30 min. The reaction was terminated by heat inactivation at 80°C for 10 min and then filtered through a 3 kDa MW cutoff filter from Millipore.

### Macrophage analysis

Primary peritoneal macrophages were collected after thioglycollate stimulation as previously described [36]. Agmatine (Sigma, 97% purity) stimulation of macrophages occurred at the specified concentrations for 24 hours in RPMI (Gibco, Life technologies) supplemented with 10% FBS, and supernatant samples were collected and analyzed for mouse TNF-α by ELISA (R&D Systems).

### NF-κB reporter mice assay

All animal studies reported in this manuscript were performed in compliance with the UMN Institutional Animal Care and Use Committee under an approved protocol. The NGL or HLL mice have been used by our lab to describe the *in vivo* activation of NF-κB in various inflammatory states [37], [38]. HLL mice were used to study the effects of intratracheal agmatine administration. Each mouse received an intratracheal injection of 100 µg agmatine in 100 µL of PBS via direct laryngoscopy while under isoflurane anesthesia. NF-κB activation at the timepoints indicated was performed as described with quantitation of luminescence over the chest using the Xenogen IVIS Spectrum *in vivo* imaging system (Caliper Life Sciences, Hopkinton, MA) for a 5 sec capture time. Each reading was normalized to the baseline luminescence of the same mouse before agmatine injection. The studies combining LPS and agmatine were performed in NGL mice as this line replaced the HLL mice in our colony during these studies. Agmatine and/or LPS were injected intraperitoneally (1 mg and 100 µg respectively in 200 µL PBS) and luminescence over the chest and abdomen was quantified and normalized on a per mouse basis.

### Mouse pneumonia model

Female BALB/C mice aged 8–12 weeks were obtained from Harlan Laboratory (Madison, WI) and used for all pneumonia studies. The infecting pseudomonads were grown to mid-log phase, washed in PBS by pelleting and resuspension, and diluted to inoculating doses after normalizing each to 10^8^ cfu/mL as estimated by OD600. Mice received the dose and strain indicated in figures via intratracheal injection in 100 µL total volume by direct laryngoscopy under isoflurane anesthesia. At the timepoints indicated mice were sacrificed, their lungs exposed and BAL was collected by tracheal puncture and instillation and return of two separate one mL volumes of PBS which were combined. Bacterial cfu in the BAL was determined by serial dilution and plating. Cell counts were determined by manual count using a hemacytometer. BAL TNF-α was determined by ELISA (R&D). Spleens were removed and homogenized before serial dilution and plating for cfu determination. Bacterial luminescence during the course of a pneumonia was detected as described for the NGL mice except IV luciferin is not required as the bacteria auto-luminesce. Detection times were 30 seconds.

### Statistical analysis

The agmatine data are approximately lognormally distributed. Due to sensitivity limitations with the UPLC-MS/MS method (LOQ  = 0.04), approximately 74% of the observations were left-censored. Data of this type are often analyzed by the substitution method, in which left-censored observations are replaced with the value LOQ or LOQ/2, and the resulting complete data set is analyzed as if there were no censoring. Although expedient, the substitution method is known to lead to biased estimates of model parameters. To minimize bias, we used a more principled technique called the method of maximum likelihood [39]. This approach has been shown to reduce bias considerably and to perform well even for censoring up to 80%. Statistics calculated using *R* (http://www.r-project.org/) and SPSS (IBM, Armonk, NY).

## Supporting Information

Figure S1 **Agmatine response in the agmatine bioluminescent reporter.** *P. aeruginosa* PA14 was constructed to the genotype Δ*speA*, *aguA:gm*, Δ*agu2ABCA*', *aguR-B:luxCDABE* which neither produces nor destroys agmatine but bioluminesces in its presence. Unfilled bars represent the reporter as described above filled bars are identical mutants missing the transcriptional element before the *luxCDABE* operon. Each bar represents the average of four wells measured 3 hours after mixing ∼1×10^6^ cfu with agmatine to a final concentration shown on the x-axis. The relative luminescence is normalized to optical density (to control for bacterial growth). Error bars represent sem. This experiment repeated >5 times with similar results.
(TIF)

Figure S2 **Agmatine augments LPS induced inflammatory response in alternate model.** The NGL mouse was used in these studies but the measurement of luminescence is the same as in Figure 5. In panel (A) mice received intraperitoneal doses of agmatine (light blue), LPS (dark blue) or agmatine and LPS (purple). There are 9 mice per group except in the Ag+LPS group in which there are 6 given 3 deaths (not analyzed). Each experiment was replicated on 2 other occasions with similar results. Panel (B) shows representative images of individual mice in these studies. Independent t-tests were used between groups of mice with relevant comparisons shown. PBS, like agmatine alone, does not induce a significant change in luminescence when injected intraperitoneally (data not shown). All error bars represent SEM. Independent t-tests used between groups. *P<0.05.
(TIF)
