# Supplementary figures and images for: The Arginine Decarboxylase Pathways of Host and Pathogen Interact to Impact Inflammatory Pathways in the Lung
Source: PLoS One. 2014 Oct 28;9(10):e111441. doi: 10.1371/journal.pone.0111441 (PMC4211729; doi:10.1371/journal.pone.0111441)

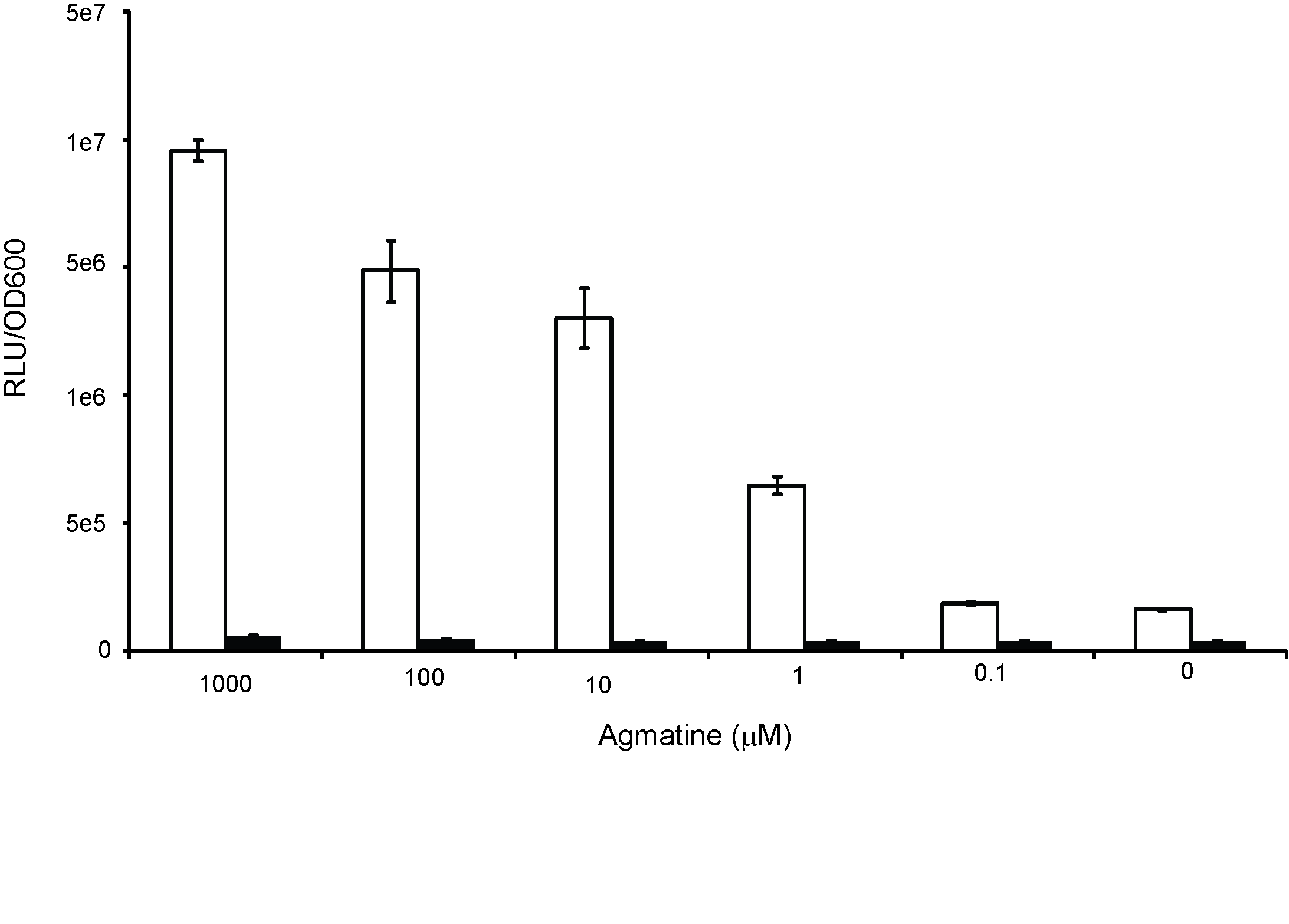

Supplement: Figure S1 — Agmatine response in the agmatine bioluminescent reporter. P. aeruginosa PA14 was constructed to the genotype ΔspeA, aguA:gm, Δagu2ABCA', aguR-B:luxCDABE which neither produces nor destroys agmatine but bioluminesces in its presence. Unfilled bars represent the reporter as described above filled bars are identical mutants missing the transcriptional element before the luxCDABE operon. Each bar represents the average of four wells measured 3 hours after mixing ∼1×10^6^ cfu with agmatine to a final concentration shown on the x-axis. The relative luminescence is normalized to optical density (to control for bacterial growth). Error bars represent sem. This experiment repeated >5 times with similar results. (TIF) [file pone.0111441.s001.tif]

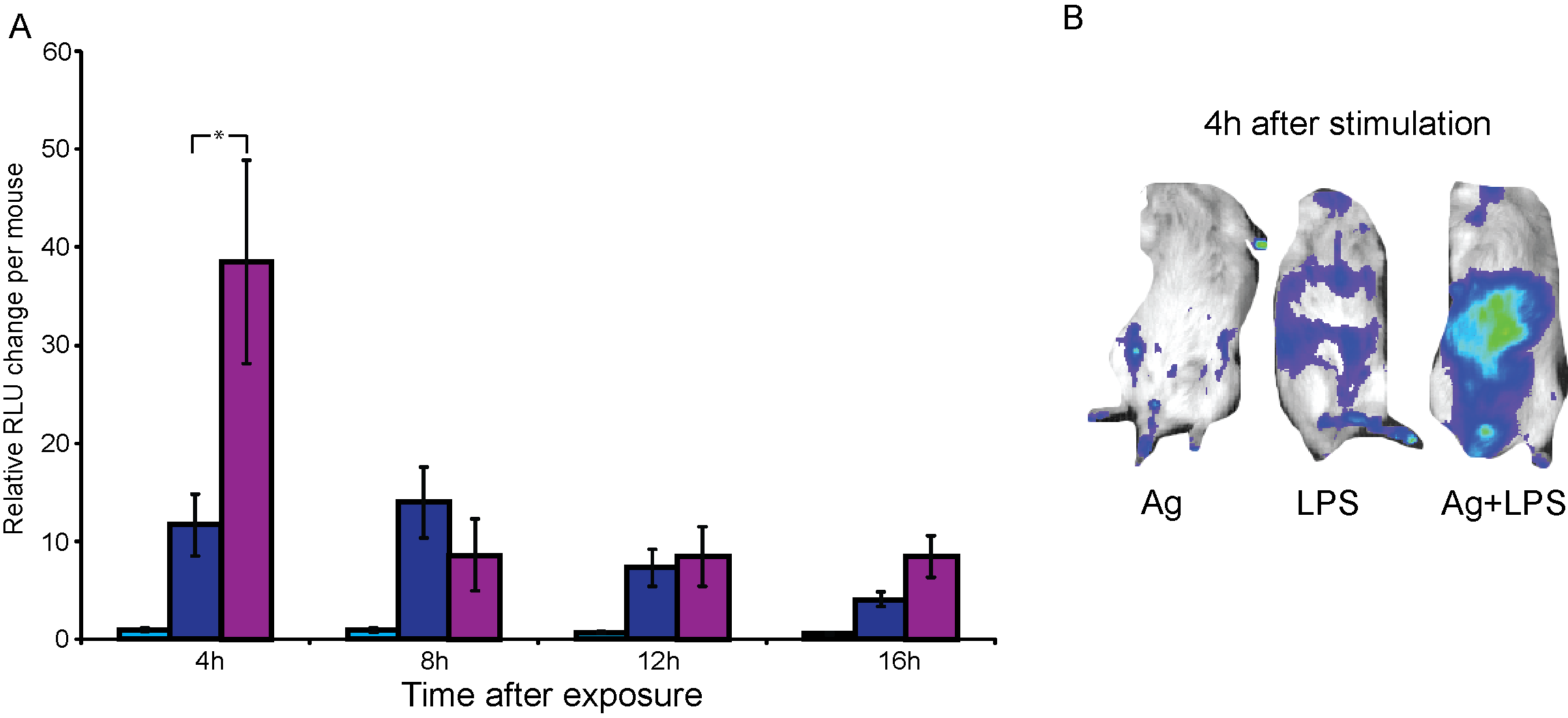

Supplement: Figure S2 — Agmatine augments LPS induced inflammatory response in alternate model. The NGL mouse was used in these studies but the measurement of luminescence is the same as in Figure 5. In panel (A) mice received intraperitoneal doses of agmatine (light blue), LPS (dark blue) or agmatine and LPS (purple). There are 9 mice per group except in the Ag+LPS group in which there are 6 given 3 deaths (not analyzed). Each experiment was replicated on 2 other occasions with similar results. Panel (B) shows representative images of individual mice in these studies. Independent t-tests were used between groups of mice with relevant comparisons shown. PBS, like agmatine alone, does not induce a significant change in luminescence when injected intraperitoneally (data not shown). All error bars represent SEM. Independent t-tests used between groups. *P<0.05. (TIF) [file pone.0111441.s002.tif]
